# Temporary Hospital Provision in War-Time

**Published:** 1914-08-29

**Authors:** 


### TEMPORARY HOSPITAL PROVISION IN WAR-TIME.

The more detailed and full the information which reaches us in regard to temporary and adapted accommodation which has been contemplated and even arranged for in many places, the more essential is it that someone in authority should call a halt. Dr. W. S. Greenfield, late professor of clinical medicine at Edinburgh, has sounded a useful note of caution by pointing out that any provision which is made for casual and semi-convalescent hospitals may be needed for some months, or even for a much longer period. If the war be relatively short the period for which these temporary hospitals will be occupied by patients must be unfortunately longer. Again, there is the further consideration that all such buildings must be suitable for occupation during winter, and this requisite, combined with that of a prolonged occupation, renders it essential that all such accommodation shall be weather-proof, and capable of being maintained at a uniform temperature during the severest weather conditions.

It would, indeed, be lamentable should the provision actually forthcoming be inadequate to receive and properly tend under the most perfect condition every wounded sailor or soldier who is sent home from the war for treatment and recovery. We are afraid that these considerations are not present in the minds of several men in responsible positions, who are doing their best to secure for the locality in which they reside an adequate hospital provision against the day of necessity. It is true, no doubt, that experience proves that hospital tents in certain circumstances can be made hygienically satisfactory, and that patients suffering from open wounds have often done exceptionally well in such conditions. We have known the successful use of tents in Russia which were even kept at an adequate temperature during the severest weather by means of a system of heating, where the furnace was kept well below the floor-level of each tent, the heat generated being arranged to circulate fully therefrom. We do not think, however, that an attempt to make any such arrangements in a temperate climate like England would justify the necessary expenditure, or be attended with equally satisfactory results. We notice, however, that at Norwich, according to a letter from the Chairman of the Board of Management of the Norfolk and Norwich Hospital to the *Eastern Daily Press*, that emergency tents are now being erected upon ground adjacent to the hospital for the reception of the wounded from the war. These tents, it is stated, have been fully equipped for the reception of patients, and can be inspected on and from the 27th instant. We gather that the scheme in question includes the provision of some 250 beds in the whole should they be required. It is noticeable, however, that the estimate of the expenditure needed to make this provision does not include, apparently, any heating arrangements, nor, if we are to judge by the prices quoted, are even the bedsteads of a quality which is high enough in our opinion for the adequate and proper reception of wounded sailors and soldiers at a time like the present. Whilst we commend the public spirit and energy displayed at Norwich and elsewhere in the direction of tents, we venture to hope that the word of caution here uttered may be taken to heart, not only in Norwich but throughout the country, whereever similar temporary provision is contemplated.

